# Association of serum AMH levels with the number of oocytes retrieved in adolescent and young adult women undergoing controlled ovarian stimulation for fertility preservation

**DOI:** 10.1007/s00404-025-07976-x

**Published:** 2025-02-18

**Authors:** Sung Woo Kim, Hee Jin Son, Ji Yeon Han, Hoon Kim, Seung-Yup Ku

**Affiliations:** 1 Department of Obstetrics and Gynecology, Seoul National University Hospital, Seoul, South Korea; 2 Department of Obstetrics and Gynecology, Seoul National University College of Medicine, 101 Daehak-ro, Jongno-gu, Seoul, 03080 South Korea; 3 Institute of Reproductive Medicine and Population, Medical Research Center, Seoul National University, Seoul, South Korea

**Keywords:** The number of oocytes retrieved, Controlled ovarian stimulation, Fertility preservation, Serum anti-müllerian hormone, Adolescent and young adult

## Abstract

**Purpose:**

To investigate whether the number of oocytes retrieved after controlled ovarian stimulation (COS) for fertility preservation (FP) can be predicted using serum anti-Müllerian hormone (AMH) levels in adolescent and young adult (AYA) women.

**Methods:**

This is a retrospective cohort study in a large university-affiliated fertility center. AYA women aged 11 to 25 years received COS using gonadotropin-releasing hormone (GnRH) antagonist protocols for FP were enrolled. Those with canceled cycles or incomplete data were excluded. The primary outcome is to determine whether the number of oocytes retrieved can be predicted through serum AMH levels using multiple linear regression analysis.

**Results:**

The mean numbers of oocytes retrieved, mature oocytes retrieved, and oocytes cryopreserved were 10.3 ± 7.6, 6.9 ± 6.2, and 8.5 ± 6.8, respectively. Multiple stepwise linear regression analysis revealed that serum AMH level independently predicts COS outcomes, including the total number of oocytes retrieved (adjusted *R*^2^ = 0.222, *P* < 0.001), the number of mature oocytes retrieved (adjusted *R*^2^ = 0.102, *P* = 0.013), and the number of oocytes cryopreserved (adjusted *R*^2^ = 0.153, *P* = 0.003).

**Conclusion:**

Serum AMH level was a significant predictor of the number of oocytes retrieved after COS for FP in AYA women, while antral follicle count and age were not significantly related.

**Supplementary Information:**

The online version contains supplementary material available at 10.1007/s00404-025-07976-x.

## What does this study add to the clinical work

**Table:** 

This study demonstrates that serum AMH level can predict the number of oocytes retrieved in adolescent and young adult (AYA) women undergoing controlled ovarian stimulation for fertility preservation. This insight enables more effective fertility preservation counseling and practice for AYA patients, allowing for the optimization of individualized protocols and improving decision-making.	

## Introduction

There are a variety of clinical situations in which fertility preservation (FP) may be considered for adolescent and young adult (AYA) women. This includes cases of AYA women diagnosed with cancer who are about to undergo chemotherapy or pelvic radiation therapy; those with non-malignant conditions such as systemic lupus erythematosus, sickle cell anemia, benign ovarian teratoma, or endometrioma, who are likely to experience a decrease in ovarian function after treatment; those with chromosomal conditions such as Turner syndrome that impair ovarian function; and transgender males with gender dysphoria [1–5]. For AYA women with cancer, it is especially important to consider quality of life, including fertility after survival, as most have not completed childbearing at the time of diagnosis and the 10-year overall survival rate is higher than 85% [6]. FP methods for women can broadly include gonadotrophin-releasing hormone (GnRH) agonist administration, oocyte cryopreservation (OC), embryo cryopreservation, and ovarian tissue cryopreservation [7]. Among these, OC is the primary method of FP for AYA women post-menarche as it can be performed without a partner, does not require an invasive surgical procedure for oocyte retrieval, and does not reintroduce malignant cells [8].

Predicting the number of oocytes retrieved has important clinical implications when considering OC in AYA women. Unlike infertile patients, AYA women considering FP may only have one chance to cryopreserve their oocytes as they are facing chemotherapy, radiotherapy, or ovarian surgery such as oophorectomy, which can reduce fertility [9]. Since the probability of having a live birth from thawed frozen oocytes through IVF is determined by the age at the time of oocyte retrieval and the number of frozen oocytes, retrieving fewer oocytes leads to fewer mature and frozen oocytes, thereby lowering the expected pregnancy rate [10]. A higher number of oocytes retrieved may increase the pregnancy rate, but it also increases the risk of developing ovarian hyperstimulation syndrome (OHSS), which can delay important treatments such as chemotherapy and surgery [11]. Therefore, predicting and providing accurate information about the number of oocytes retrieved during FP counseling in AYA women is critical for clinicians, patients, and their caregivers to decide whether to pursue FP through OC.

Anti-Müllerian hormone (AMH) is a peptide hormone produced by the granulosa cells of preantral and early antral follicles in the ovary [12]. Serum AMH levels remain relatively constant between and within menstrual cycles, making it the gold-standard ovarian reserve test for assessing ovarian function [13]. Numerous studies have demonstrated the usefulness of serum AMH levels in predicting ovarian response and the number of oocytes retrieved during controlled ovarian stimulation (COS) in infertile patients undergoing in vitro fertilization (IVF), or in adult patients with breast cancer undergoing oocyte or embryo cryopreservation for FP [14, 15]. However, there are no studies on whether serum AMH levels are helpful in predicting ovarian response and the number of oocytes retrieved during COS for OC in AYA women who need FP. Therefore, this study aims to analyze the relationship between serum AMH levels and the number of oocytes retrieved in AYA women who undergo COS for FP, and to determine whether the number of oocytes retrieved can be predicted through serum AMH levels.

## Materials and methods

### Data source and patient population

Data were collected from the medical records of women referred to the FP clinic at Seoul National University Hospital between January 2020 and October 2022. Women who underwent COS using GnRH antagonist protocols for FP, aged 11 to 25 years at the time of oocyte retrieval, are included. The exclusion criteria are canceled cycles and cycles with incomplete data.

### Study variables and outcomes

Baseline demographics and clinical data included age at the time of oocyte retrieval, body mass index (BMI, kg/m^2^), antral follicle count (AFC), serum AMH level (ng/mL), basal serum follicle-stimulating hormone (FSH) level (mIU/mL), types of COS, starting dose and total dose of gonadotropins (IU), duration of stimulation, and serum estradiol and progesterone levels (pg/mL) on human chorionic gonadotropin (hCG) day. Patients with OHSS according to the Golan criteria [16] were also assessed.

AFC (average diameter of 2–10 mm) and basal serum FSH level were measured on menstrual cycle day (MCD) 2 or 3 for conventional COS cycles, and on stimulation day 1 for random start COS cycles. Conventional COS was conducted according to the GnRH antagonist protocol. Recombinant FSH (Gonal-F, Serono) was initiated on MCD 2 or 3, and treatment was maintained with the addition of the GnRH antagonist cetrorelix (Cetrotide 0.25 mg, Serono) when the leading follicle reached 14 mm in diameter. Recombinant hCG (Ovidrel 250 mcg, Serono) was administered 36 h prior to oocyte retrieval when the leading follicle reached 18 mm or when two or more follicles reached 17 mm in diameter. For the random start protocol, COS was initiated regardless of the menstrual cycle and was otherwise identical to the GnRH antagonist protocol. Mature oocytes (metaphase II, MII) retrieved during ovum pick-up were cryopreserved immediately, while immature oocytes (germinal vesicle, GV, and metaphase I, MI) were observed for up to 24 h, and if they reached the MII stage, they were cryopreserved.

### Statistical analyses

Baseline characteristics and COS outcomes were presented as means and standard deviations. Student’s *t*-test and Chi-square test were used to compare means and proportions between groups, respectively. Univariate linear regression and multiple stepwise linear regression were used to analyze predictive factors for COS outcomes. *P* values less than 0.05 were considered statistically significant. All data analyses were performed using the Statistical Package for Social Science 12.0 (SPSS Inc., Chicago, IL, USA) and R Statistical Software (R Foundation for Statistical Computing, Vienne, Austria, Version #4.3.0).

## Results

### General characteristics of the study population

**Fig. 1:**
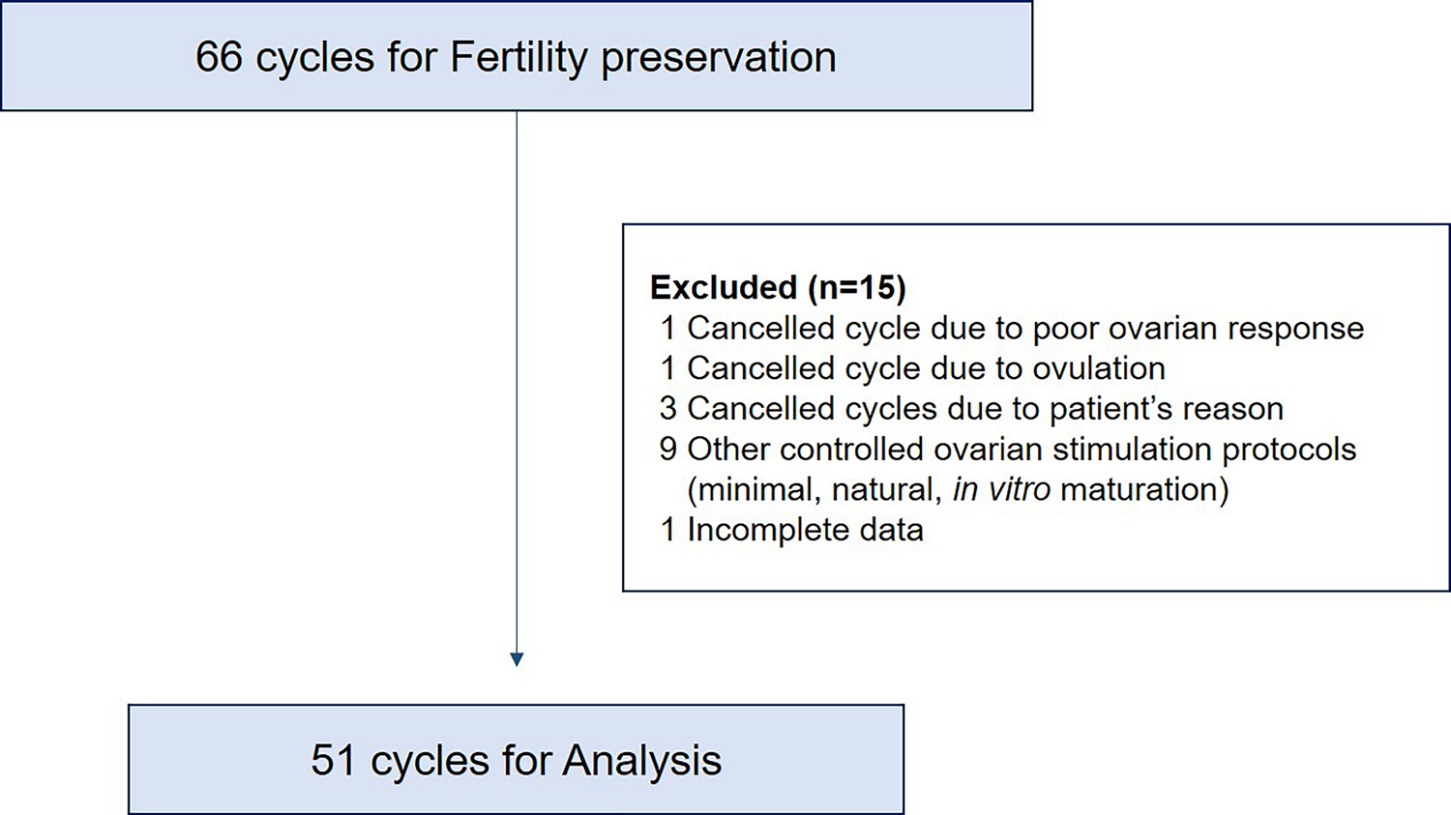
Flowchart of the study population

A total of 66 cycles were conducted in our clinic for FP, of which 51 that followed the antagonist protocols were analyzed (Fig. 1). The baseline characteristics of these patients are summarized in Table 1. The mean age of the analyzed patients was 19.3 years. 33.3% of the patients underwent fertility preservation due to malignant diseases, including hematologic and solid tumors, while the remaining 66.7% had non-malignant reasons such as benign diseases, aplastic anemia, and chronic granulomatous diseases. The mean AFC and serum AMH levels were 12.1 and 2.8 (ng/mL), respectively. Of the 51 cycles, 35 underwent conventional COS and 16 underwent random start COS.

**Table 1:** Baseline characteristics of the study participants (*n* = 51)

Characteristic	Value
Age (year)	19.3 ± 4.1
BMI (kg/m^2^)	22.9 ± 4.1
AFC (*n*)	12.1 ± 11.3
Serum AMH level (ng/mL)	2.8 ± 2.8
Basal serum FSH level (mIU/mL)	5.1 ± 3.1
Type of COS	
Conventional COS (%)	35 (68.6%)
Random start stimulation (%)	16 (31.4%)
Starting dose of gonadotropins (IU)	313 ± 124
Duration of stimulation (days)	8.7 ± 3.0
Total dose of gonadotropins (IU)	2765 ± 1422
Serum estradiol level on hCG day (pg/mL)	1756 ± 1131
Serum progesterone level on hCG day (pg/mL)	0.99 ± 0.71

Data are expressed as mean ± standard deviation or *n* (%)

*BMI* body mass index; *AFC* antral follicle count; *AMH* anti-müllerian hormone; *FSH* follicle stimulating hormone; *COS* controlled ovarian stimulation; *hCG* human chorionic gonadotropin

### COS outcomes

The mean starting and total doses of gonadotropins were 313 IU and 2765 IU. The mean numbers of oocytes retrieved, mature oocytes retrieved, and oocytes cryopreserved were 10.3, 6.9, and 8.5, respectively (Table 2). No statistically significant difference was observed in the numbers of oocytes retrieved, mature oocytes retrieved, and oocytes cryopreserved between adolescents (under 18 years old) and young adults (18 years old or older) (*P* = 0.668, *P* = 0.690, and *P* = 0.569, respectively). Among young adult patients over the age of 18, one experienced OHSS and another had an infection.

**Table 2:** COS outcomes and related complications

COS outcomes and related complication	Value			
	All age (*n* = 51)	Adolescent (*n* = 18)	Young adult (*n* = 33)	*P* value^a^
Number of oocytes retrieved	10.3 ± 7.6	9.76 ± 7. 4	10.6 ± 7	0.668^t^
Number of mature oocytes retrieved	6.9 ± 6.2	6.4 ± 5.2	7.2 ± 0. 6.8	0.690^t^
Number of oocytes cryopreserved	8.5 ± 6.8	7.7 ± 6.0	8.9 ± 7.3	0.569^t^
OHSS	1 (2.0%)	0 (0%)	1 (3.0%)	1.000^f^
Bleeding	0	0	0	–
Infection	1 (2.0%)	0 (0%)	1 (3.0%)	1.000^f^

Data are expressed as mean ± standard deviation or *n* (%). Adolescent: aged under 18 years old; Young adult: aged 18 years old or older

*COS* controlled ovarian stimulation; *OHSS* ovarian hyperstimulation syndrome

^a^Between the adolescent (< 18 years old) and the young adult (≥ 18 years old)

^t^*t*-test

^f^Chi-square test

### AMH is a predictive factor of the number of oocytes retrieved, mature oocytes retrieved, and oocytes cryopreserved

Age, BMI, and traditional ovarian reserve markers (AFC, AMH, and basal FSH) are established factors correlated with oocyte retrieval outcomes in adult population. We analyzed the relationship between these factors and COS outcomes using linear regression in AYA women (Supplementary Table 1). Simple linear regression demonstrated that AFC is significantly correlated with the number of oocytes retrieved and cryopreserved (both *P* < 0.05). Serum AMH levels showed strong correlations with the number of oocytes retrieved (*P* < 0.001), mature oocyte retrieved (*P* < 0.05), and oocyte cryopreserved (*P* < 0.01). The starting dose of gonadotrophin, which was decided considering the weight of patients, ovarian reserve markers, and response in previous cycles, was also strongly correlated with the number of oocytes retrieved (*P* < 0.01), mature oocytes retrieved (*P* < 0.05), and oocytes cryopreserved (*P* < 0.01).

**Fig. 2:**
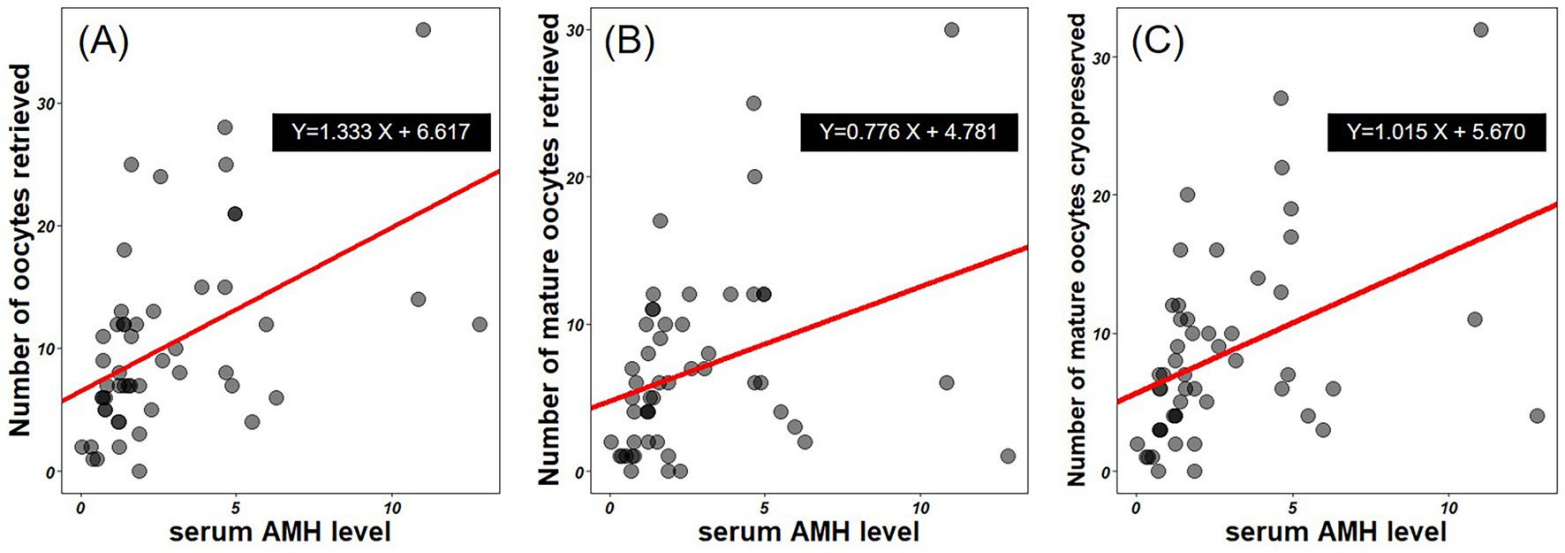
Multiple stepwise linear regression models evaluating the relationship between serum AMH levels and controlled ovarian stimulation outcomes. (**A**) Number of oocytes retrieved, (**B**) number of mature oocytes retrieved, (**C**) number of mature oocytes cryopreserved. The analyzed variables included age, body mass index, serum AMH level, basal serum FSH level, and starting dose of gonadotropins. AMH: anti-Müllerian hormone; FSH: follicle-stimulating hormone

Multiple stepwise linear regression analysis, including factors age, BMI, AMH, basal FSH level, and starting dose of gonadotropins, identified only serum AMH level as an independent factor predicting COS outcomes (Table 3, Fig. 2). Serum AMH level was predictive of **t**he total number of oocytes retrieved (adjusted *R*^2^ = 0.222, *P* < 0.001), the number of mature oocytes (adjusted *R*^2^ = 0.102, *P* = 0.013), and the number of oocytes cryopreserved (adjusted *R*^2^ = 0.153, *P* = 0.003). When age, BMI, AFC, basal FSH level, and starting dose of gonadotropins were included in the regression analysis, the starting dose of gonadotropins was found to be a statistically significant factor, whereas the conventional ovarian reserve marker AFC was not significant (Supplementary Table 2).

**Table 3:** Multiple stepwise linear regression analyses between variables of baseline characteristics and COS outcomes, including variables: age, BMI, AMH, basal serum FSH level, and starting dose of gonadotropins

	Number of oocytes retrieved			Number of mature oocytes retrieved			Number of mature oocytes cryopreserved		
	*B*	*P*	Adjusted *R*^2^	*B*	*P*	Adjusted *R*^2^	*B*	*P*	Adjusted *R*^2^
Age	– 0.003	0.978	0.222	– 0.029	0.831	0.102	0.015	0.910	0.153
BMI	0.059	0.643		0.103	0.449		0.087	0.508	
Serum AMH level	1.333	< 0.001		0.776	0.013		1.015	0.003	
Basal serum FSH level	0.021	0.870		0.156	0.251		0.064	0.632	
Starting dose of gonadotropins	– 0.168	0.333		– 0.196	0.292		– 0.239	0.185	

*COS* controlled ovarian stimulation; *BMI* body mass index; *AMH* anti-müllerian hormone; *FSH* follicle stimulating hormone

## Discussion

FP methods for women can be broadly categorized into GnRH agonist administration, OC, embryo cryopreservation, and ovarian tissue cryopreservation [7]. FP for AYA women should be individualized based on factors including age, pubertal development, underlying medical conditions or comorbidities, life expectancy, and religious beliefs [17]. The fertility-preserving effects of GnRH agonist administration are unclear except for breast cancer, and GnRH agonist does not offer ovarian protection during pelvic radiation therapy [18]. Embryo cryopreservation may not be feasible as a primary option for AYA women without a partner. Ovarian tissue cryopreservation can be performed on girls before menarche but involves invasive surgery to obtain ovarian tissue, and there are concerns about the efficacy and safety in terms of live birth rates and the potential reintroduction of cancer cells [19]. In vitro follicular maturation is an alternative, although optimal culture conditions have not yet been established [20, 21]. Recently, there have been attempts to regenerate female reproductive organs and restore damaged fertility using tissue engineering methods; however, numerous challenges must be overcome before clinical application becomes feasible [22]. In contrast, OC using vitrification does not require invasive surgery and is currently the primary FP method considered for AYA women post-menarche because it avoids the concern of reintroducing cancer cells [8].

This is the first study to analyze the association between serum AMH levels and the number of oocytes retrieved during COS for FP in AYA women, and to determine if serum AMH levels can predict the number of oocytes retrieved. In this study, only serum AMH levels were significantly associated with predicting the number of oocytes retrieved after COS for FP in AYA women, whereas AFC and age were not significant predictors. This finding suggests that serum AMH levels can be used to predict which patients will have an optimal number of oocytes retrieved, those likely to exhibit a poor response, and potential complications such as OHSS, making it a useful counseling tool for FP in AYA women considering OC. As most AYA women have only one opportunity to retrieve oocytes, and complications such as OHSS can delay subsequent treatments such as chemoradiotherapy or surgery, it is important to retrieve as many oocytes as possible at once without complications [9, 11]. According to the results of this study, the expected number of oocytes retrieved is serum AMH level ×1.333 + 6.617. This indicates that for every 1 ng/mL increase in serum AMH level, 1.3 more oocytes are expected to be retrieved. Clinically, predicting ovarian response is critical for FP counseling prior to COS for OC. Poor ovarian response is related to cost-effectiveness, including the possibility of empty follicles and how many COS cycles will be needed to cryopreserve the desired number of oocytes. High ovarian response is related to the safety of COS, i.e., the possibility of delaying subsequent treatment for the underlying disease due to complications such as OHSS.

The mean number of oocytes retrieved in the present study was 10.3, which is comparable to previous studies reporting on OC for FP in AYA women [3, 23, 24]. However, most of the previous studies are case reports or case series with no control group and small sample sizes. A large retrospective study was conducted, but it only described the outcomes of OC in adolescent women up to age 20, compared to adult women, and the use of national data from the SART CORS database means that there is no data on patient-specific factors such as ovarian reserve markers and weight, so the number of oocytes retrieved is presented without consideration of these factors [25]. Another retrospective cohort study also had the disadvantage of including 9 out of 38 patients with a history of chemotherapy that caused a significant decrease in ovarian function, and did not predict the number of oocytes retrieved [26]. There is also a study on OC in women with a history of childhood and adolescent cancer. However, this study has limitations in that it included not only AYA women but also adult women, COS was conducted after chemotherapy, and the number of patients was small [27].

The mean age of participants included in the present study was 19.3 years, making postmenarchal AYA women the primary focus of this cohort study. The rate of cycle cancelation due to poor ovarian response and the incidence of OHSS after COS were both 1 of 51 (2.0%) in this study, which are not high compared to rates observed in the adult population [25]. There were no cases of bleeding after the oocyte retrieval procedure. Using multiple stepwise linear regression to control confounding variables, neither AFC nor age significantly predicted the number of oocytes collected, contrasting with findings in the adult population. The reduced predictive value of age in AYA women may stem from differences in maturation and activation of the hypothalamic-pituitary-ovarian axis, with the age of menarche being a more critical determinant of ovarian response than chronological age [28]. In AYA women, serum AMH levels do not decrease with age and exhibit a different longitudinal trajectory compared to adult women [29]. It is also important to note that the women in this study were not healthy but had been diagnosed with cancer or ovarian diseases, suggesting that the general condition or extent of ovarian damage, rather than age alone, might influence ovarian response. Women in poor general health due to cancer or ovarian diseases like endometriosis are known to have poorer response to COS [30, 31].

In adults, both serum AMH levels and AFC as markers of ovarian reserve are known to be good predictors of the number of oocytes retrieved [32]. However, in this study, only serum AMH levels were significantly associated with predicting the number of oocytes retrieved; while AFC was not a significant predictor. Several factors may contribute to this conclusion. First, the present study assessed AFC primarily using transabdominal or transrectal ultrasound rather than transvaginal ultrasound, which may have reduced accuracy [33]. In adult women, AFC is most often assessed by transvaginal ultrasound, but in this study, AFC was assessed using transabdominal or transrectal ultrasound, not transvaginal ultrasound due to concerns about hymenal injury. Considering the potential inaccuracy of transabdominal ultrasound in assessing AFC, the existing literature on OC in AYA women has not employed transabdominal ultrasound to measure AFC, but only to monitor ovarian response during COS [3, 34].

Second, some patients had their AFC measured during the luteal phase rather than the early follicular phase, as random start stimulation accounted for 31.4% of all cycles in the present study. Unlike infertile patients who typically wait until the early follicular phase of the next cycle to begin COS, patients considering FP often cannot afford to delay treatment. Therefore, random start stimulation protocols, which do not depend on the menstrual cycle phase, are frequently used to initiate COS promptly [35]. When AFC is measured during the luteal phase, the corpus luteal cyst formed after ovulation can obscure the AFC, which ranges from 2 to 10 mm in diameter, thereby complicating accurate assessment [36]. Although AFC can be measured at any point during the menstrual cycle, it is advisable to measure it during the early follicular phase to minimize inaccuracies [37, 38].

This study analyzed AYA women who underwent oocyte retrieval and cryopreservation via conventional COS at a single center. Future studies should evaluate the predictive model of this study through both internal and external validation. The AYA women in this study are more likely to have a benign rather than a malignant condition as the reason for undergoing oocyte retrieval. In addition, patients who cryopreserved their oocytes through minimal stimulation, natural cycle, or in vitro maturation (IVM) rather than conventional COS were excluded. Given the variations in age distribution, reasons for OC, and the COS protocols among different studies, caution should be exercised when generalizing and applying the results of this study to all AYA women who undergo COS for FP.

The study found that serum AMH levels were an independent predictor of the number of oocytes retrieved in AYA women undergoing COS for FP. Starting doses of gonadotropins for COS were often determined without knowing serum AMH levels, as the decision to cryopreserve oocytes for FP in AYA women was frequently made under urgent circumstances. Based on the findings of this study, it is advisable to perform serum AMH test in AYA women as soon as a disease affecting fertility is diagnosed. In addition, there is a need to develop various tools, in addition to serum AMH levels, that can predict ovarian response and the number of oocytes retrieved in AYA women for more detailed counseling. Recent studies have utilized artificial intelligence to predict the number of oocytes retrieved by synthesizing factors such as age, AFC, and BMI along with AMH in adults, yet research in this area remains scarce for AYA women [39].

A large body of data has accumulated on OC in adult cancer patients, and a recent meta-analysis confirmed the live birth rate after thawing cryopreserved oocytes in cancer survivors [40]. However, the number of AYA women who have undergone OC remains relatively small compared to adult women [25], and their young age complicates the analysis of live birth rates due to the extended period before pregnancy and childbirth. To accurately assess outcomes related to live birth, it is essential to establish a prospective cohort of AYA women who require OC. This cohort should be developed through close collaboration among multiple centers to continuously gather and analyze data, including post-thaw fertilization rates, live birth rates, and cumulative live birth rates over an extended period. We hope that our findings will inspire further research in this field and assist fertility specialists and AYA women considering OC in making informed medical decisions.

In conclusion, this study suggests that serum AMH hormone levels, not age or antral follicle count, could predict the number of oocytes retrieved after controlled ovarian stimulation in adolescents and young adults. This insight has enabled more effective fertility preservation counseling and practice for adolescents.

## Supplementary Information

Below is the link to the electronic supplementary material.

Supplementary file1 (DOCX 24 KB)

## Data Availability

The datasets used during the current study are available from the corresponding author on reasonable request.

## Abbreviations

AFC: Antral follicle count
AMH: Anti-müllerian hormone
AYA: Adolescent and young adult
BMI: Body mass index
COS: Controlled ovarian stimulation
FP: Fertility preservation
FSH: Follicle-stimulation hormone
GnRH: Gonadotropin-releasing hormone
hCG: Human chorionic gonadotropin
IVF: In vitro fertilization
IVM: In vitro maturation
MCD: Menstrual cycle day
OC: Oocyte cryopreservation
OHSS: Ovarian hyperstimulation syndrome
