# Supplementary material for: Association of serum AMH levels with the number of oocytes retrieved in adolescent and young adult women undergoing controlled ovarian stimulation for fertility preservation
Source: Arch Gynecol Obstet. 2025 Feb 18;311(3):811–8. doi: 10.1007/s00404-025-07976-x (PMC11920294; doi:10.1007/s00404-025-07976-x)
Supplement: Supplementary file 1 — Supplementary file1 (DOCX 24 KB) [file 404_2025_7976_MOESM1_ESM.docx]

**Supplementary Table S1.**

Simple linear regression analyses between variables of baseline characteristics and COS outcomes

|  | **Number of oocytes retrieved** | **Number of mature oocytes retrieved** | **Number of mature oocytes cryopreserved** |
| --- | --- | --- | --- |
| Age | 0.056 ± 0.265 | -0.008 ± 0.217 | 0.072 ± 0.238 |
| BMI | 0.071 ± 0.262 | 0.132 ± 0.214 | 0.117 ± 0.236 |
| AFC | 0.234 ± 0.090* | 0.131 ± 0.077 (P=0.095) | 0.183 ± 0.083* |
| Serum AMH level | 1.333 ± 0.341*** | 0.776 ± 0.301* | 1.015 ± 0.321** |
| Basal serum  FSH level | -0.090 ± 0.346 | 0.225 ± 0.282 | 0.031 ± 0.312 |
| Starting dose of gonadotropins | -0.026 ± 0.008** | -0.017 ± 0.007* | -0.023 ± 0.007** |

Data are expressed as regression coefficient ± standard error, * *P*<0.05, ** *P*<0.01, *** *P*<0.001

COS: controlled ovarian stimulation; BMI: body mass index; AFC: antral follicle count; AMH: anti-

müllerian hormone; FSH: follicle stimulating hormone

**Supplementary Table S2.**

Multiple stepwise linear regression analyses between variables of baseline characteristics and COS outcomes, including variables: age, BMI, AFC, basal serum FSH level, and starting dose of gonadotropins

|  | Number of oocytes retrieved | | | Number of mature oocytes retrieved | | | Number of mature oocytes cryopreserved | | |
| --- | --- | --- | --- | --- | --- | --- | --- | --- | --- |
|  | *B* | *P* | Adjusted  R^2^ | *B* | *P* | Adjusted  R^2^ | *B* | *P* | Adjusted  R^2^ |
| Age | 0.004 | 0.977 | 0.163 | -0.026 | 0.847 | 0.098 | 0.018 | 0.892 | 0.150 |
| BMI | 0.028 | 0.831 |  | 0.080 | 0.557 |  | 0.060 | 0.650 |  |
| AFC | 0.101 | 0.578 |  | -0.001 | 0.995 |  | 0.034 | 0.853 |  |
| Basal serum  FSH level | -0.039 | 0.765 |  | 0.111 | 0.412 |  | 00.012 | 0.929 |  |
| Starting dose of gonadotropins | -0.026 | 0.002 |  | -0.017 | 0.014 |  | -0.023 | 0.003 |  |

COS: controlled ovarian stimulation; BMI: body mass index; AFC: antral follicle count; FSH: follicle stimulating hormone
